# Correlation between socioeconomic indices and epidemiological indices of thyroid cancer from 1990 to 2019 year: a global ecologic study

**DOI:** 10.1186/s12885-024-12176-y

**Published:** 2024-04-15

**Authors:** Zahra Maleki, Jafar Hassanzadeh, Haleh Ghaem

**Affiliations:** 1 Student Research Committee, Shiraz University of Medical Sciences, Shiraz, Iran; 2 Department of Epidemiology, School of Health, Shiraz University of Medical Sciences, Shiraz, Iran; 3 Non-Communicable Diseases Research Center, Department of Epidemiology, School of Health, Shiraz University of Medical Sciences, Shiraz, Iran

**Keywords:** Thyroid neoplasms, Incidence, Mortality, Ecological study, World

## Abstract

**Background:**

The incidence of thyroid cancer as the most common type of endocrine gland malignancy has risen more significantly than any malignancies in recent years. Estimated new cases of thyroid cancer in the United States in 2024 were 12,500 and 31,520 for men and women, respectively, and estimated deaths were 1,180 for women and 990 for men. Indices of socio-economic have been commonly used to measure the development of countries. Therefore, this study aimed to examine the correlation between indices of socioeconomic status and epidemiological indices of thyroid cancer throughout the world. In addition, this study has compared two indices of human development and a socio-demographic index.

**Method:**

This worldwide ecological study used data on thyroid cancer incidence, mortality, human development index (HDI), and sociodemographic index (SDI) between 1990 and 2019 from the Global Burden of Disease (GBD). We evaluated the correlation between incidence and mortality rates with socioeconomic indices by using Pearson’s correlation coefficient. Furthermore, for the first time, the generalized additive model (GAM) was employed for modeling. The statistical software R, version 4.2.2, was used to conduct all statistical analyses.

**Results:**

The correlation between the incidence of thyroid cancer and the HDI was significant and positive (*r* = 0.47, p-value < 0.001). While the correlation between thyroid cancer mortality and HDI was not statistically significant (*r* = 0.01, p-value = 0.076). Besides, the incidence of thyroid cancer was significantly positively correlated with SDI (*r* = 0.48, p-value < 0.001). The multiple GAM showed that for one unit increase in HDI, the risk of thyroid cancer was increased by 2.1 times (RR = 2.1, 95%CI = 2.04 to 2.19), and for one unit increase in SDI, the risk of thyroid cancer was shown to increase by 2.2 times. (RR = 2.2, 95%CI = 2.19 to 2.35).

**Conclusion:**

It has been evident that countries with higher incidence of thyroid cancer display higher socioeconomic indices. While, countries with higher socioeconomic indices, report lower mortality rates. However, based on the modeling results, it can be concluded that the SDI is slightly more useful in this regard. Therefore, examining the epidemiological indices of thyroid cancer by socio-economic indices can be useful to reflect a clear image of the distribution of this cancer in each country, and can be used for planning cancer prevention strategies.

**Supplementary Information:**

The online version contains supplementary material available at 10.1186/s12885-024-12176-y.

## Introduction

Thyroid cancer is indeed the most common endocrine malignancy and the fifth most common type of cancer among women. The incidence of thyroid cancer has risen more significantly than any malignancies in recent years [1]. Estimated new cases of thyroid cancer in the United States in 2024 were 12,500 and 31,520 for men and 31,520, respectively, and estimated deaths were 1,180 for women and 990 for men [2]. Thyroid cancer has a comparatively low mortality rate [3]. Early-stage thyroid cancer is about 4 times more common in women than in men, but the underlying subclinical prevalence is the same between the sexes [4]. The incidence of this cancer has been elevated in all continents, while this trend was not shown to increase in the African continent, which may be due to lack of cancer diagnosis [5]. The mortality rate due to early diagnosis remains constant at around 1–2% [6]. The thyroid cancer incidence in all age groups is 3 to 4 times higher in women than men [7, 8]. The thyroid cancer incidence rate in developed countries is 2-fold higher in both genders compared to developing countries [9]. The incidence of this cancer steadily varies around the world, which may depend on reasons, such as access to medical care, racial and ethnic differences, geographic and environmental variations, including iodine excess or iodine deficiency, and radiation exposure [10].

The Human Development Index (HDI) is a useful measure of a country’s social and economic development, based on three key dimensions: health, education, and income. The health index is determined by a country’s life expectancy at birth, while the education index is based on adult literacy rates, combined gross enrollment rates in primary, secondary, and higher education, and compulsory education duration. The income index is calculated using the country’s GDP per capita PPP adjusted by purchasing power parity and total population (US dollars) http://hdr.undp.org/en/composite/IHDI [11, 12].

Multiple pathologies, including cancer, are associated with these indices [13]. The relationship between these indices and the incidence and mortality rates of cancer has been investigated in various studies. Still, the results vary depending on the location of the study and the type of cancer being examined [14–18].

Studying the impact of living conditions, reflected in a composite index like HDI, on the epidemiology of thyroid cancer in the world is necessary to determine what public health measures should be taken. Additionally, knowledge of the incidence and mortality rates of thyroid cancer can be useful for health programs and research activities, and considering the potential role of HDI can be valuable. Therefore, this study aimed to determine the correlation between socioeconomic indices (i.e., HDI & SDI) and epidemiological indices (i.e., incidence and mortality rates) of thyroid cancer. This study not only explored the relationship between HDI and thyroid cancer, but it also examined the correlation between SDI and this type of cancer. Previous studies had mainly focused on the correlation with HDI, and there were limited studies that investigated the relationship with SDI. The primary aim of this study was to determine the correlation between the top socioeconomic index and the epidemiological indices of thyroid cancer.

## Methods

This global ecological study examined the correlation between thyroid cancer incidence and mortality rates with HDI and SDI. Incidence and mortality data for all patients in each year for 204 countries and regions between 1990 and 2019 were obtained from the Global Burden of Disease (GBD) (available at https://www.healthdata.org/research-analysis /gbd) [19]. Data on HDI was obtained by the 2021/2022 Human Development Report for every country in the world.

The SDI is a tool that gauges the development level of countries or geographic regions by taking into account various factors such as per capita income, educational attainment, and fertility rates. The measure is expressed on a scale of 0 to 1 and is calculated by averaging the rankings of these variables across all the areas included in the GBD study. This index combines information regarding the economic, education, and fertility rates of countries all around the world by highlighting social and economic development, and health outcome variables are closely correlated with this index. The data on SDI from 1990 to 2019 is available at https://ghdx.healthdata.org/record/ihme-data/gbd-2019-socio-demographic-index-sdi-1950-2019 [20].

### Statistical analyses

Descriptive statistics (mean and standard deviation) were used to summarize the quantitative data. The correlation between epidemiological indices (i.e., incidence and mortality rates) and socioeconomic position indices (HDI & SDI) was assessed using Pearson’s correlation coefficient. The strength of agreement assessed by McBride’s criteria as follows: poor, < 0.90; moderate, 0.90–0.95; substantial, 0.95–0.99; almost perfect > 0.99 [21]. Besides, due to the lack of a linear relationship between epidemiological indices and socioeconomic indices, the generalized additive model (GAM) was employed to determine this association. The GAM model was used to estimate the relative risk (RR) of the effect of socioeconomic Indices on the incidence and mortality of thyroid cancer. This model is an extended form of the generalized linear model (GLM) and has high flexibility. This model has been used in many studies because it can adjust nonlinear confounding parameters. In this research, variables with a correlation of 0.2 greater than the main prediction were included in the model. The time unit used in the analysis was the year. Using the formula RR = Exp (β), we calculated the relative risk and 95% confidence interval = exp (β ± 1.96 SE) for RR [22–24]. Statistical analysis was performed using R and “mgcv” packages [24]. A p-value < 0.05 was considered statistically significant. All reported p-values were two-tailed.

## Results

Descriptive information on epidemiological indices of thyroid cancer and socioeconomic indices are shown in Table 1. The mean incidence and mortality of thyroid cancer were 2.398 and 0.647, respectively. Also, the mean HDI and SDI were 0.647 and 0.562, respectively.

**Table 1:** Descriptive characteristics of epidemiological indexes of thyroid cancer and socioeconomic indexes

Variables	Mean ± standard deviation	P25	P50	P75	Minimum	Maximum
Incidence rate per 100,000	2.398 **±** 1.938	0.937	1.854	3.393	0.054	22.437
Mortality rate per 100,000	0.647 **±** 0.403	0.407	0.546	0.773	0.031	4.714
HDI	0.647 **±** 0.167	0.517	0.671	0.774	0.168	0.973
SDI	0.562 **±** 0.192	0.426	0.581	0.711	0.050	0.929

### HDI

**Fig. 1:**
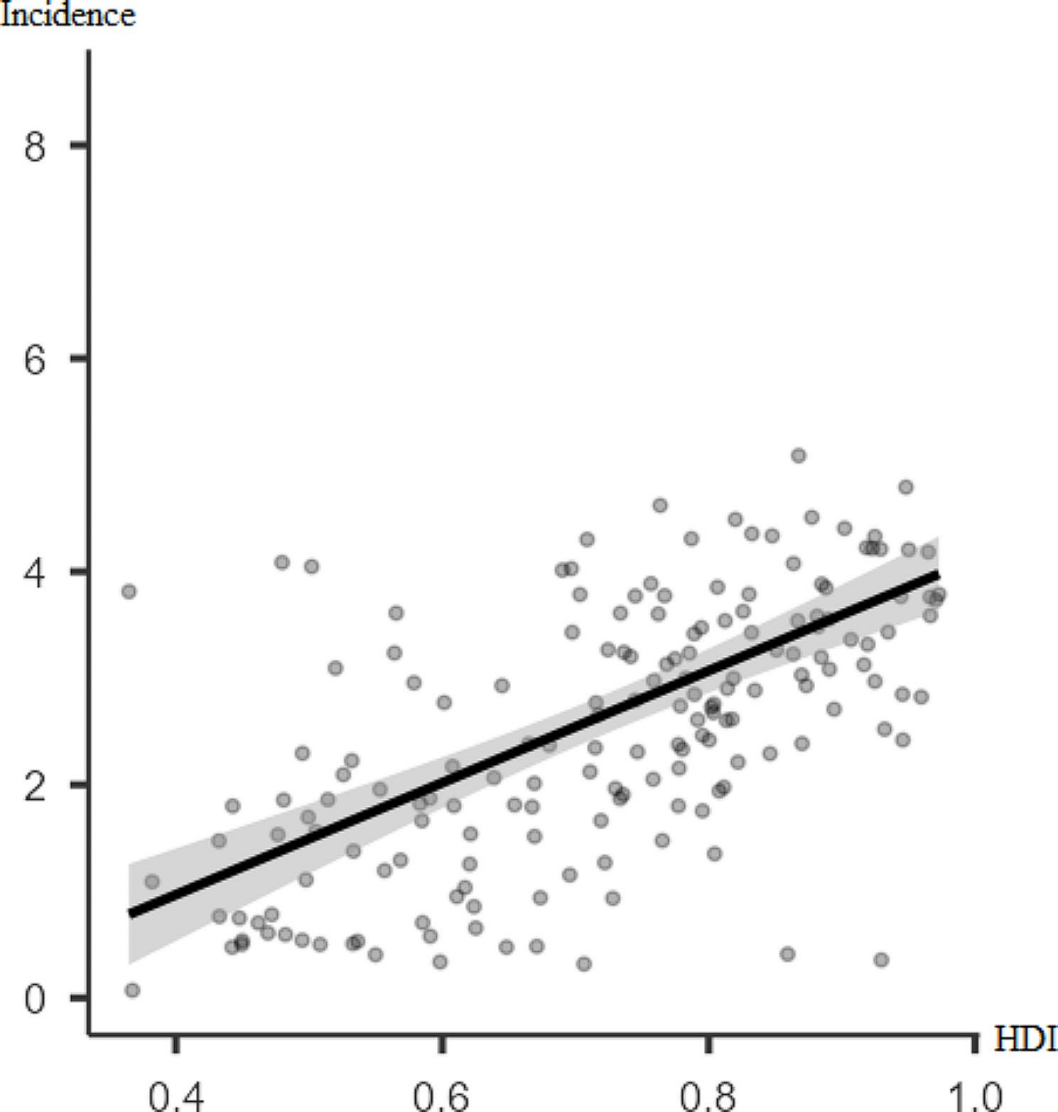
Pearson correlation between the incidence rate of thyroid cancer for both sexes in 2019 and HDI

**Fig. 2:**
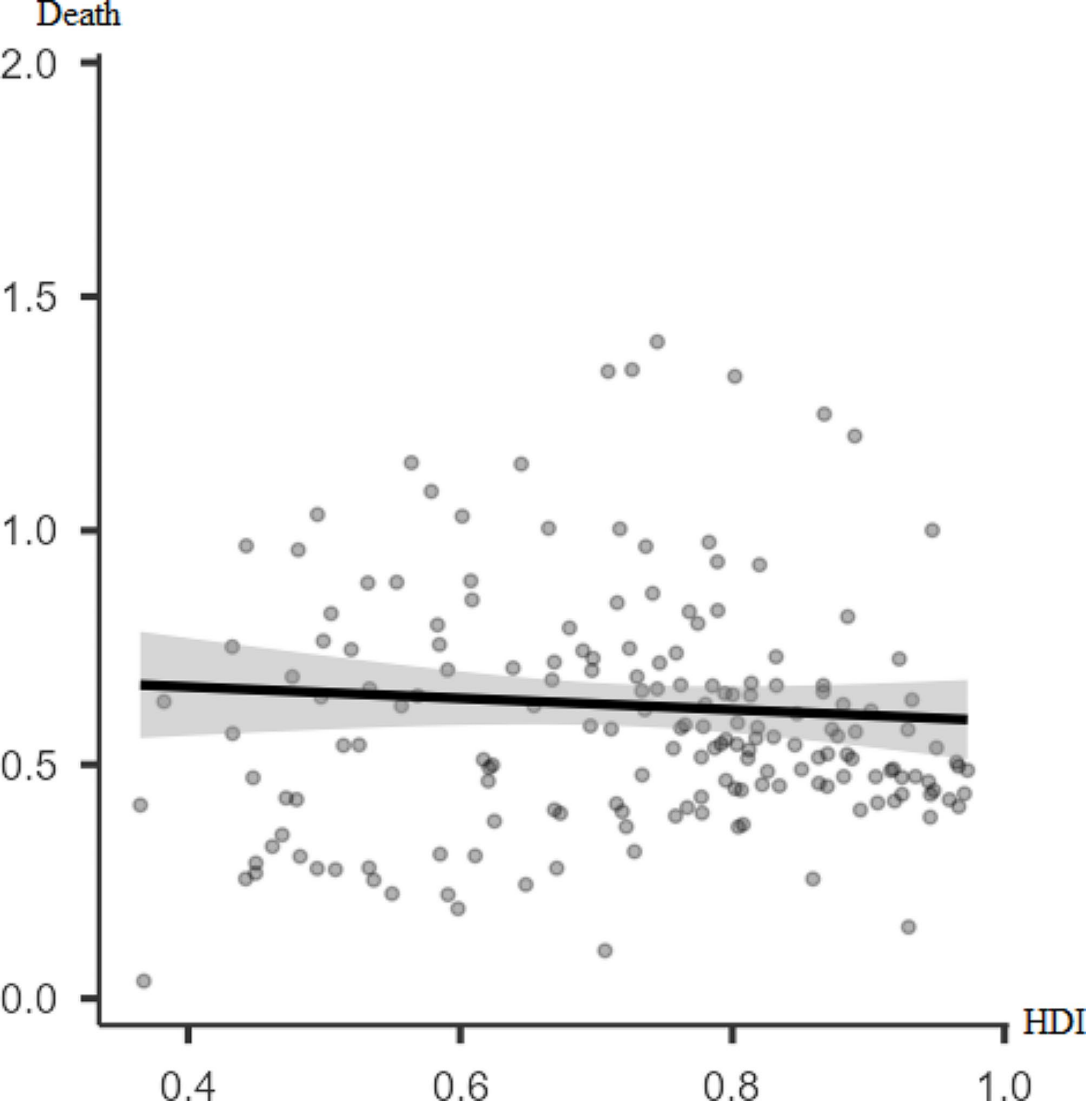
Pearson correlation between the mortality rate of thyroid cancer for both sexes in 2019 and HDI

According to Figs. 1 and 2, there is a significant positive poor correlation between the incidence rate of thyroid cancer and HDI (*r* = 0.47, p-value < 0.001). However, there was no statistically significant correlation between thyroid cancer mortality rate and HDI (*r* = 0.01, p-value = 0.076).

The results of the multiple GAM model showed that one unit increase in HDI, increased the risk of thyroid cancer by 2.1 times. While one unit increase in HDI, decreased the risk of thyroid cancer mortality by 9% (Table 2).

**Table 2:** Multiple GAM for relationship between HDI and incidence/ mortality rates of thyroid cancer

Variable	Incidence rate					Mortality rate				
	β	RR	SE	95% CI	P-value	β	RR	SE	95% CI	P-value
HDI	0.75	2.13	0.018	2.04 to 2.19	< 0.0001	-0.09	0.91	0.004	0.90 to 0.92	< 0.0001

### SDI

**Fig. 3:**
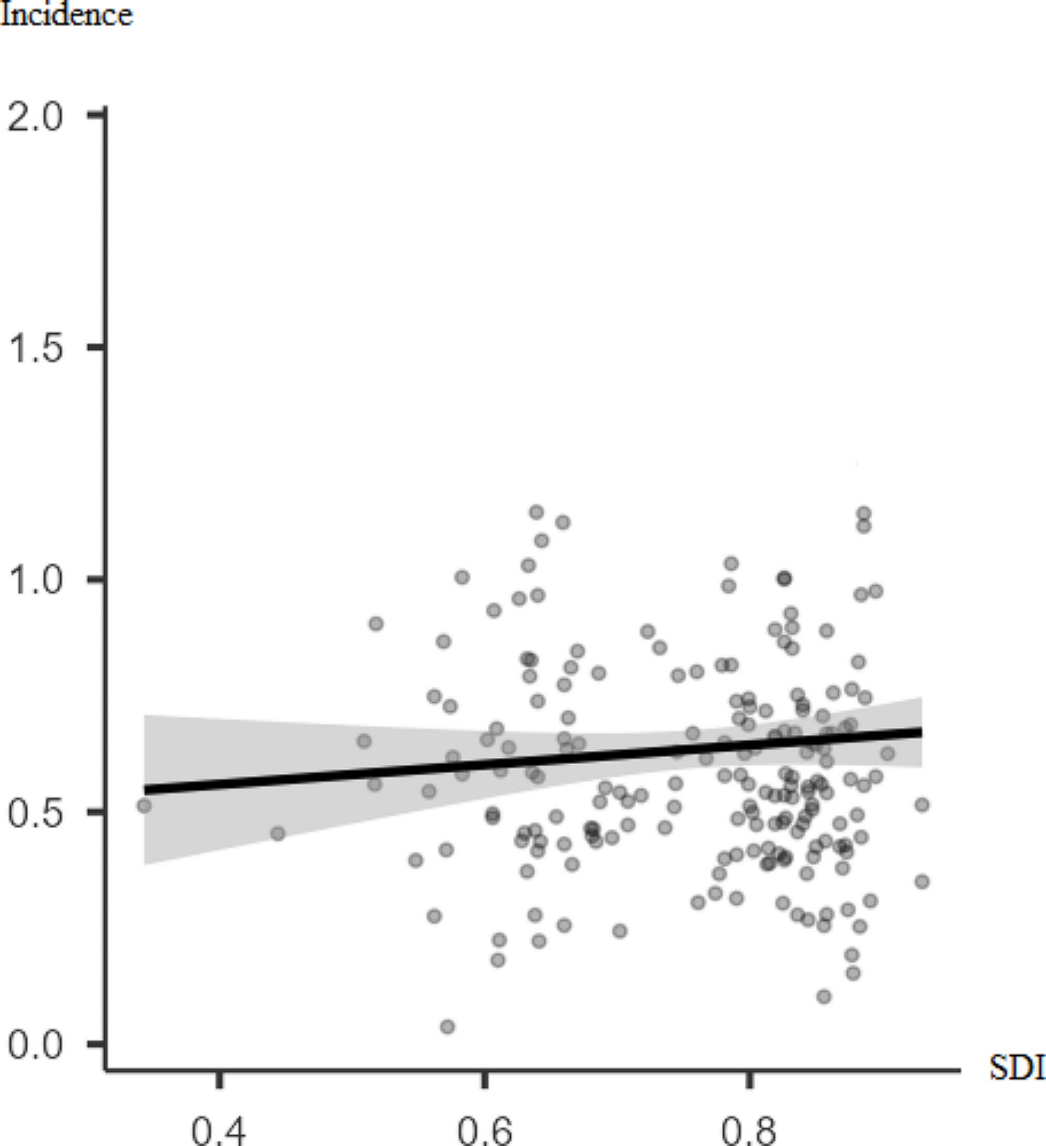
Pearson correlation between the incidence rate of thyroid cancer for both sexes in 2019 and SDI

**Fig. 4:**
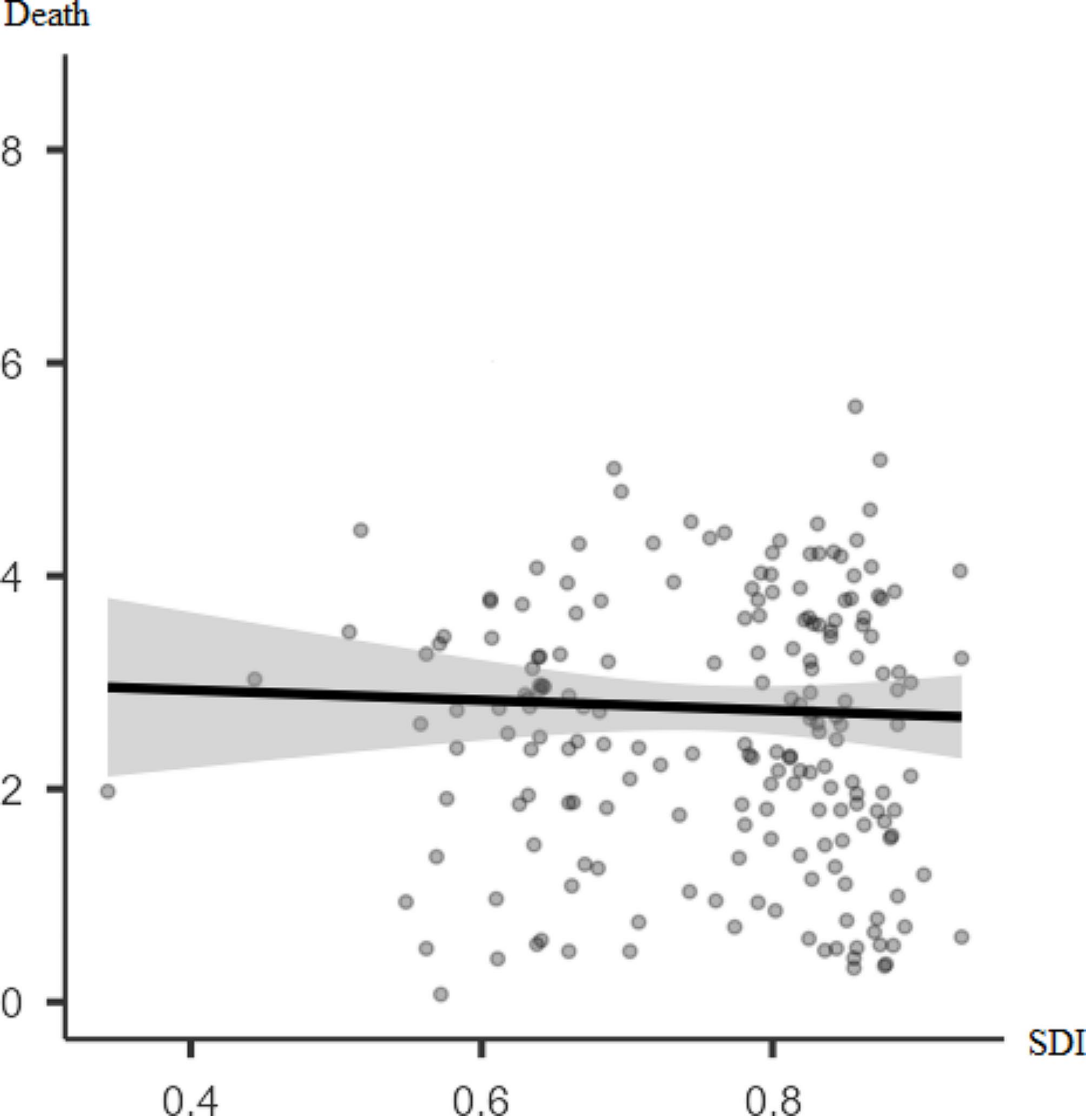
Pearson correlation between the mortality rate of thyroid cancer for both sexes in 2019 and SDI

The incidence rate of thyroid cancer is significantly positively poor correlated with SDI (*r* = 0.48, p-value < 0.001). However, there was no significant correlation between thyroid cancer mortality rate and SDI (*r* = 0.003, p-value = 0.719) (Figs. 3 and 4).

The results of the multiple GAM model showed that for one unit increase in SDI, the risk of thyroid cancer was increased by 2.2 times, whereas one unit increase in SDI, decreased the risk of thyroid cancer mortality by 11% (Table 3).

**Table 3:** Multiple GAM for relationship between SDI and incidence/ mortality rates of thyroid cancer

Variable	Incidence rate					Mortality rate				
	β	RR	SE	95% CI	P-value	β	RR	SE	95% CI	P-value
SDI	0.82	2.27	0.018	2.19 to 2.35	< 0.0001	-0.11	0.89	0.004	0.88 to 0.90	< 0.0001

The geographical distribution of the incidence and mortality rate of this cancer for both sexes in 2019 is also presented in Figures S5 and S6 (Figs S1 and S2).

## Discussion

This study was conducted to determine the correlation between socioeconomic indices and epidemiological indices of thyroid cancer all over the world. In the present global ecological study, we found the following: Firstly, the results of the present study showed a significant positive correlation between the human development index and thyroid cancer incidence. Also, the modeling results illustrated that an increased risk of incidence of thyroid cancer is associated with elevated HDI. Secondly, increased HDI is correlated with a decreased mortality rate of this cancer. Thirdly, the findings of this study showed a significant positive correlation between the incidence of thyroid cancer and SDI. In addition, results of GAM showed that one unit increase in SDI, increased the risk of thyroid cancer by 2.2 times. Fourthly, for one unit increase in SDI, the risk of thyroid cancer mortality rate decreased by 11%.

### HDI

These findings were supported by Goodarzi et al. (2019) [10]. Ghoncheh and colleagues conducted a study titled “Breast Cancer Incidence and Mortality and Its Relation with global HDI in 2012”, and concluded a significant positive correlation between age-standardized incidence rate (ASIR) and HDI components [25]. This may be due to the density of endocrinologists and general surgeons and the use of neck ultrasound, which explained 57% of the variation in state-level incidence rates for men and 49% for women [26]. A significant portion of thyroid cancer cases diagnosed in high-resource countries in the past two decades is likely due to diagnostic changes. This ratio has gradually increased over time and is expected to continue increasing in the future. While there is evidence of harm, there is no evidence of benefit from thyroid screening. Therefore, the risks associated with excessive diagnosis and overtreatment of thyroid cancer should be immediately addressed [27]. Areas with the highest utilization of optional medical tests (neck ultrasound), highest population density, and better education had the highest rate of thyroid cancer diagnosis. The difference in the rate of ordering optional diagnostic medical tests, such as diagnostic ultrasound, in different geographical areas of Ontario leads to differences in the rate of thyroid cancer diagnosis [28]. The doubling increase in thyroid cancer incidence is associated with an approximately 5-fold increase in thyroid ultrasound utilization and an approximately 7-fold increase in fine needle aspiration thyroid biopsy between the years 2000 and 2012 [29]. Molecular imaging has become a key technology for personalized medicine due to its high efficiency and minimal side effects in monitoring molecular changes, cellular processes, and the microenvironment of tumors. Thyroid cancer is the most common malignancy of the endocrine glands, and radioiodine therapy is widely used for the diagnosis or treatment of differentiated thyroid cancer [30]. Also, Iodine-131 (I-131) is a radiopharmaceutical that can be used for diagnostic and therapeutic purposes. The therapeutic goal includes treating thyroid malignancy in patients after surgery and hyperthyroidism. Well-differentiated thyroid carcinomas respond well to I-131 treatment. This activity highlights the role of an interdisciplinary healthcare team in evaluating and treating patients for better clinical outcomes [31]. According to the SEER database, one of the main reasons for the increase in thyroid cancer diagnoses was advanced diagnosis, which is evident in developed countries. Improved diagnosis probably contributed to the increase in thyroid cancer incidence in the past decades, but it cannot fully explain the increase and suggests that there is a real increase. It is imperative to ascertain the underlying cause for this observed rise [32].

In 2019, Herrera-Serna and colleagues investigated the association between the HDI and its components with oral cancer in Latin America. Their findings indicate that in countries with medium and low HDI, HDI was inversely correlated with oral cancer mortality [14]. Another study also concluded that the highest incidence of thyroid cancer occurred in very high-HDI countries and the highest mortality rates were observed in low- and medium-HDI countries [6]. Therefore, the improvement of diagnostic and treatment methods in high-HDI countries, the industrial lifestyle, and increased exposure to the risk factors of this cancer, including medical radiation, ionizing radiation, cosmic radiation, pesticides, and carcinogenic solvents, may play a role in the relationships found [20]. Other risk factors for thyroid cancer that have been identified in both developed and developing countries include exposure to certain chemicals during early life, autoimmune thyroid disease, overweight and obesity, alcohol consumption, and smoking. In general, higher HDI in countries is associated with a higher incidence but lower mortality rate of this cancer [10]. The 10-year survival rate of thyroid cancer is between 80 and 95% based on the type of carcinoma. This cancer is diagnosed at different ages. Higher age at diagnosis is linked with a lower cancer survival rate. Therefore, in countries with lower developmental status, diagnosis occurs later, and subsequently, death rate is higher in these countries. However, the death rate will be lower in developed countries [6, 25]. Considering that one of the components of the HDI is life expectancy at birth, thus, in developed countries with higher HDI, increased life expectancy, is associated with elevated duration of exposure to the risk factors of this cancer. The result of a study also showed that life expectancy at birth is directly related to a higher risk of cancer [6]. Also, Yang and colleagues in Germany showed that the survival of patients with thyroid cancer sharply decreased at the age of 60 years [33].

### SDI

In line with the results of our study, YuJiao Deng and colleagues evaluated the global burden of thyroid cancer from 1990 to 2017 and demonstrated that the incidence of thyroid cancer was positively correlated with SDI, while the mortality rate of this cancer was significantly inversely correlated with SDI [34]. Another study conducted in 204 countries in 2021 also showed that the global incidence of this cancer continued to increase over the last three decades. One of the most important risk factors associated with thyroid cancer in countries with high SDI is elevated body mass index (BMI) [35]. Azadnajafabad et al. performed a systematic review in 2021 and showed that countries with higher socioeconomic status displayed the highest incidence and lowest mortality rates of thyroid cancer, which complied with the results of our study. This is probably due to better access to health care and early diagnosis in these countries [36]. Previous studies have shown that the increased incidence of this cancer can be caused by the socioeconomic status and the level of development of a society. For example, the prevalence of thyroid cancer in African men (79 per 100,000 people) significantly differs from Australian men (365 per 100,000 people) [10]. A study in Los Angeles showed that countries with high socioeconomic levels reported higher incidence of thyroid cancer than countries with low socioeconomic status. It is also possible that the main cause of the increase in the metastases of this cancer is the delay in providing medical and health care, which can have reducing effects on the prognosis of this cancer. Therefore, the prognosis of this cancer is more favorable in developed countries with better economic status compared to developing countries [6]. Finally, low socioeconomic status is associated with more advanced papillary thyroid cancer at presentation and lower adjuvant radioiodine rates after total thyroidectomy, especially among patients younger than 45 years from areas with moderate household income [37]. Also, a study was conducted to investigate the practice of radioactive iodine therapy and clinical-social factors related to radioactive iodine dose in differentiated thyroid cancer patients among Asian countries. This study showed Different radioactive iodine dose ranges are used in the low-risk group probably because the physicians consider radioactive iodine dose elevation based on clinical-social factors beyond pre-existed guidelines [38]. Also, the influence of ethnic and geographic diversity on distinct outcomes of thyroid cancer is well documented and shows distinct and different observations in different countries. Age at diagnosis, gender, risk classification, and tumor stage influence disease progression and mortality. Survival rates were favorable for patients who received radioactive iodine treatment. In addition, adherence to institutional clinical practice guidelines leads to better disease control. Studies on the Middle Eastern population show that those residing in developing countries have a slightly lower survival rate when compared to developed countries [39]. Consistent with the results of the present study, black patients and those with low socioeconomic status have worse outcomes for thyroid cancer. Asian-Pacific Islander and Hispanic patients may have a protective effect on survival despite having more advanced diseases [40, 41].

### Strengths and limitations of the study

One of the strengths of this global study is the examination and comparison of two indices that determine socioeconomic status. Also, this study for the first time examined the modeling of socioeconomic indices with epidemiological indices of thyroid cancer. The weakness of this study is the use of aggregate data. Therefore, readers may fall into an ecological fallacy in interpreting the findings. This is one of the limitations of ecological studies, so ecological studies are hypothesis-generating.

## Conclusion

The main objective of this study was to identify the correlation between the best socioeconomic index and thyroid cancer epidemiological indices. Based on the modeling results, it can be concluded that the SDI indices are slightly more useful in this regard. However, the HDI Index focuses more on health and education, while the SDI indices emphasize socioeconomic status. The Human Development Index is still an important Index for investigating the social and health status of communities and can be used as a general Index for examining the health status of a community. Both the Human Development Index and the socio-demographic Index are important tools used to assess the social and health status of communities.

### Electronic supplementary material

Below is the link to the electronic supplementary material.

Supplementary Material 1

Supplementary Material 2

## Data Availability

Data AvailabilityThe data that support the findings of this study are available from the corresponding author, [HGH], upon reasonable request.
