# Supplementary figures and images for: Correlation between socioeconomic indices and epidemiological indices of thyroid cancer from 1990 to 2019 year: a global ecologic study
Source: BMC Cancer. 2024 Apr 15;24:467. doi: 10.1186/s12885-024-12176-y (PMC11017482; doi:10.1186/s12885-024-12176-y)

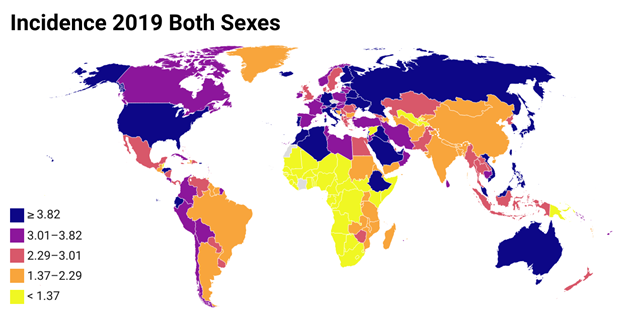

Supplement: Supplementary file 1 — Supplementary Material 1 [file 12885_2024_12176_MOESM1_ESM.tif]

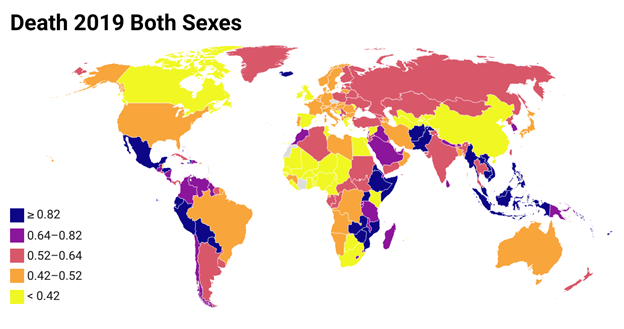

Supplement: Supplementary file 2 — Supplementary Material 2 [file 12885_2024_12176_MOESM2_ESM.tif]
